# Arterial catheterization and in-hospital mortality in sepsis: a propensity score-matched study

**DOI:** 10.1186/s12871-022-01722-5

**Published:** 2022-06-09

**Authors:** Qitian Ou, Gengxin Cai, Yuan Zhou, Wanjie Zha, Linqiang Huang, Hongke Zeng, Wenqiang Jiang, Shenglong Chen, Miaoyun Wen

**Affiliations:** 1 The Second School of Clinical Medicine, Southern Medical University, Guangzhou, Guangdong China; 2 Department of Emergency and Critical Care Medicine, Guangdong Provincial People’s Hospital, Guangdong Academy of Medical Sciences, 106 Zhongshan Er Road, Guangzhou, 510080 Guangdong China; 3 Department of Critical Care Medicine, Guangdong Provincial People’s Hospital’s Nanhai Hospital, The Second People’s Hospital of Nanhai District Foshan City, Foshan, Guangdong China

**Keywords:** Arterial catheterization, Sepsis, In-hospital mortality

## Abstract

**Background:**

Despite the extensive use of arterial catheterization (AC), clinical effectiveness of AC to alter the outcomes among patients with sepsis and septic shock has not been evaluated. The purpose of this study is to examine the association between the use of AC and in-hospital mortality in septic patients.

**Methods:**

Adult patients with sepsis from Medical Information Mart for Intensive Care database were screened to conduct this retrospective observational study. Propensity score matching (PSM) was employed to estimate the relationship between arterial catheterization (AC) and in-hospital mortality. Multivariable logistic regression and inverse probability of treatment weighing (IPTW) were used to validate our findings.

**Results:**

A total of 14,509 septic patients without shock and 4,078 septic shock patients were identified. 3,489 pairs in sepsis patients without shock and 589 pairs in septic shock patients were yielded respectively after PSM. For patients in the sepsis without shock group, AC placement was associated with increased in-hospital mortality (OR, 1.34; 95% CI, 1.17–1.54; *p* < 0.001). In the septic shock group, there was no significant difference in hospital mortality between AC group and non-AC group. The results of logistic regression and propensity score IPTW model support our findings.

**Conclusions:**

In hemodynamically stable septic patients, AC is independently associated with higher in-hospital mortality, while in patients with septic shock, AC was not associated with improvements in hospital mortality.

**Supplementary Information:**

The online version contains supplementary material available at 10.1186/s12871-022-01722-5.

## Background

Arterial catheterization (AC) is commonly used in intensive care units (ICU) for invasive estimation of blood pressure (BP) [1]. It is believed to be more accurate and reliable than noninvasive BP measurements in shock states, allowing continuous measurement of BP and facilitating arterial blood gases monitoring [2–4].

However, several potential complications might occur during the use of AC. First, it is a cause of bloodstream infection, whose overall incidence is 1.7 per 1000 catheter days, higher than peripheral venous access [5]. Besides, its localized complications are common, such as limb ischemia and hematoma [6, 7]. Furthermore, it not only comes with increased costs and prolonged ICU stay but also leads to more frequent phlebotomy [8].

Despite the widespread use of AC, high-quality research focusing on the effect of AC on patients with sepsis and septic shock is still absent [9]. In view of the low complication occurrence rate and seemingly higher accuracy, the Surviving Sepsis Campaign guideline (2021) issued a weak recommendation for using invasive BP monitoring in sepsis [10]. However, whether the benefits of AC outweigh the risks remains uncertain [9].

This cohort study aims to examine the association between AC use and outcomes in septic patients with/without shock using propensity score matching (PSM) analysis. We hypothesized that AC would bring more harm than benefit to patients with sepsis.

## Methods

### Study population

We conducted this retrospective observation study of adult patients from the fourth edition of Medical Information Mart for Intensive Care database (MIMIC IV, version 1.0) [11]. MIMIC is a large, publicly available single-center critical care database housing deidentified health-related data of 382,278 individuals and 321,406 adults from years 2008 to 2019 admitted to the Beth Israel Deaconess Medical Center in Boston, Massachusetts. In our study, adult patients with sepsis (defined by sepsis 3.0 criteria) were screened [12]. The following inclusion and exclusion criteria were used: (1) only the first ICU admission of each patient was included; (2) patients with evidence of infection (antimicrobials and blood culture) and organ dysfunction (SOFA score 2 +) were included; (3) patients with length of hospital stay more than 24 h were included; (4) only patients without cardiac surgery were included. Patients were divided into the sepsis without shock group and septic shock group based on the hemodynamic status (also defined by sepsis 3.0 criteria). Patients who had AC placement after ICU admission were categorized as the AC group, while patients without the use of AC making up the non-AC group.

### Covariates and outcomes

The following available variables (without significant missing data) were extracted from MIMIC-IV database for the first day of ICU admission: age, gender, weight, admission type, ethnicity, first care unit, Sequential Organ Failure Assessment (SOFA) score, Simplified Acute Physiology Score II (SAPII), Charlson Comorbidity Index, congestive heart failure (CHF), renal disease, chronic pulmonary diseases (COPD), malignancy, liver disease, heart rate, temperature (℃), mean arterial pressure (MAP), white blood cell (WBC) count, hemoglobin, hematocrit, platelet, sodium, potassium, bicarbonate, chloride, blood urea nitrogen (BUN), creatine, glucose, anion gap, international normalized ratio (INR), prothrombin time (PT), active partial thromboplastin time (APTT), receipt of continuous renal replacement therapy (CRRT), mechanical ventilation (MV), and sedative medication. For repeated measurements, only the first result was used. And comorbid conditions were identified based on International Classification of Disease, the Ninth Version (ICD9) and Tenth Version (ICD10) [13].

We observed that the proportion of missing data of the above covariates was less than 10% (Figure S1), thus for those who have the laboratory results during hospitalization, the earliest result from ICU admission were used, and the mean imputation was used while there was no related laboratory result.

### Statistical analysis

Continuous variables were presented as mean (SD) and categorical variables were presented as number (percentage). T-test and Chi-square (χ2) were used to analyze continuous and categorical variables, respectively.

Three distinct analytical approaches were used to estimate the relationship between AC and hospital mortality. First, propensity score matching (PSM) was conducted to balance the baseline characteristics between the AC group and non-AC group. We used a logistic regression model with all covariates listed above to calculate the propensity score for each patient. Pairs were matched without replacement on the logit of the propensity score, and a nearest-neighbor 1:1 matching scheme with a caliper size of 0.2 was applied for all matched pairs. After matching, standardized mean differences (SMD) were used to evaluate the balance of baseline characteristics between the matched groups. If SMD > 0.1, the variable can be considered imbalance between the AC group and non-AC group. The odds ratios (OR) and their 95% CIs, and *p*-values were calculated for each model. Bonferroni correction was performed for the 38 *p*-values within these models, including AC/non-AC, 34 noncategorical independent variables, 3 indicator variables for the independent variable of initial ICU type. After adjusted for this multiplicity, *p*-value less than 0.0013(0.05/38) were considered significant. Besides, we performed the above analysis in patients with AC placed within 24 h after ICU admission.

### Sensitivity analysis

We conducted a series of sensitivity analyses to evaluate the robustness of the finding of the study. We used different modeling methods, multivariable logistic regression and inverse probability of treatment weighting (IPTW) analyses, to access the association between hospital mortality and AC after adjustment for the covariates. In subgroups analysis, we repeated the multivariable logistic regression in each stratified subgroup separately according to age, gender, SOFA score, and the use of MV, CRRT and sedative medication.

## Results

**Fig. 1:**
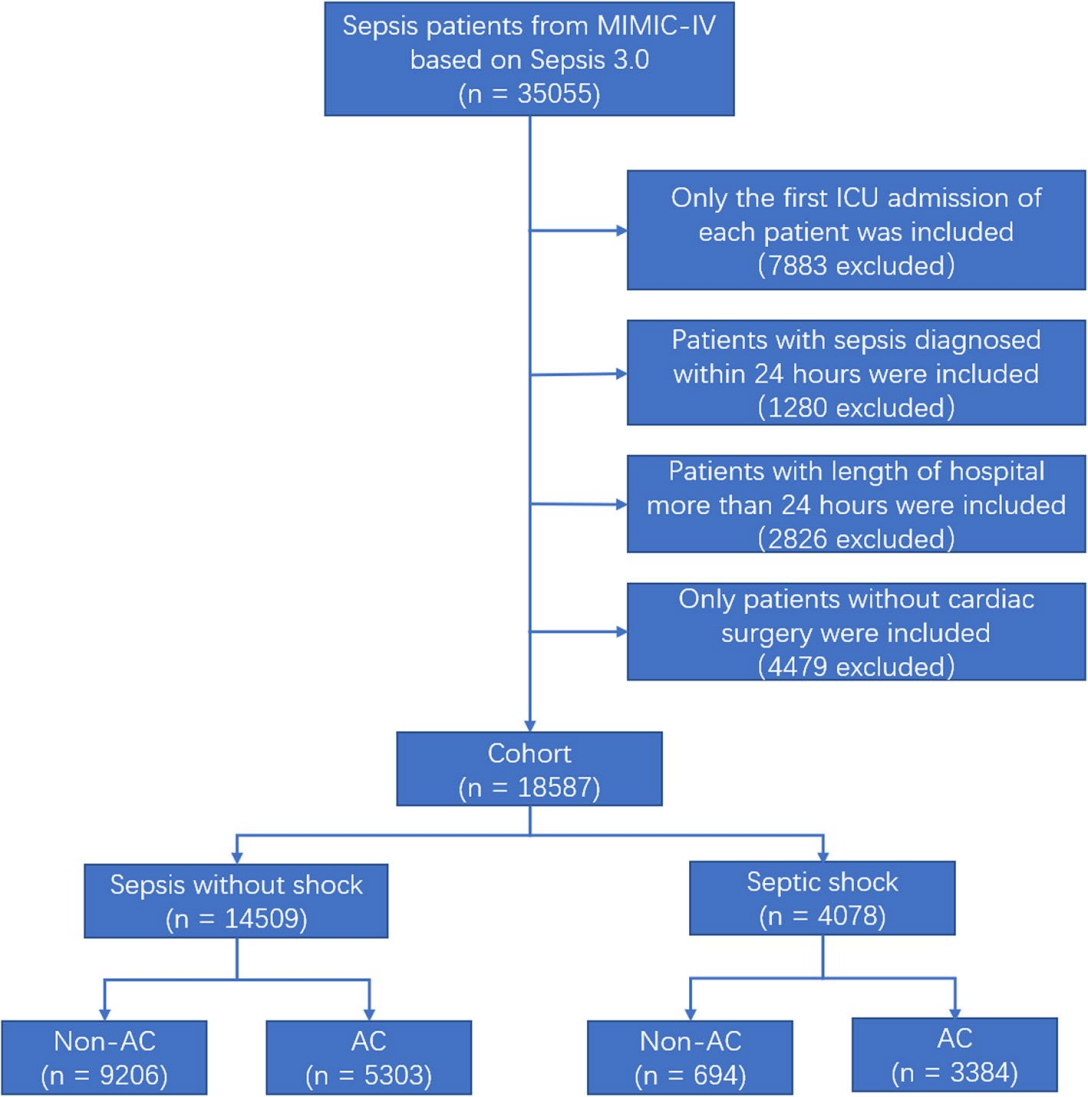
Patient Selection Flowchart. AC, arterial catheterization

After reviewing 35,055 adult patients with sepsis from MIMIC-IV, a total of 14,509 sepsis patients without shock and 4,078 septic shock patients met the inclusion and exclusion criteria (Fig. 1). Among these patients, 5,303 (36.6%) sepsis patients without shock used AC, while 3,384 (83.0%) septic shock patients used AC. Table 1 summarizes the baseline characteristics of the cohort. In both septic shock and sepsis without shock groups, the AC patients were younger, in more severe condition (SOFA score 4.35 vs 4.14 in septic shock group and 3.18 vs 3.13 in sepsis without shock group), and had a higher percentage of CRRT, MV, and sedative medication. In addition, the length of ICU stay (8.29 vs 4.93 in septic shock group and 7.56 vs 3.44 in sepsis without shock group, both *p* < 0.001) and hospital stay (16.57 vs 12.70 in septic shock group and 15.71 vs 10.85 in sepsis without shock group, both *p* < 0.001) of AC group were also longer than those of non-AC group.

**Table 1:** Baseline Characteristics Between AC and Non-AC Groups in Original Cohorts and Propensity-Matched Cohorts

Covariate	Septic shock—original cohort			Septic shock—matched cohort			Sepsis without shock—original cohort			Sepsis without shock—matched cohort		
	**Non-AC (*N* = 694)**	**AC (*N* = 3,384)**	**SMD**	**Non-AC (*N* = 589)**	**AC (*N* = 589)**	**SMD**	**Non-AC (*N* = 9,206)**	**AC (*N* = 5,303)**	**SMD**	**Non-AC (*N* = 3,489)**	**AC (*N* = 3,489)**	**SMD**
	Mean (SD)	Mean (SD)		Mean (SD)	Mean (SD)		Mean (SD)	Mean (SD)		Mean (SD)	Mean (SD)	
Age	**68.56 (16.50)**	**63.77 (16.07)**	**0.294**	67.89 (16.86)	67.81 (15.68)	0.005	**67.46 (17.29)**	**63.74 (16.62)**	**0.219**	64.86 (17.67)	64.82 (16.55)	0.002
Weight	**78.54 (21.63)**	**84.96 (25.04)**	**0.274**	78.90 (21.11)	78.97 (20.32)	0.003	**79.22 (23.65)**	**84.79 (25.27)**	**0.227**	82.14 (24.64)	82.41 (23.48)	0.011
SOFA score	**4.14 (2.12)**	**4.35 (2.43)**	**0.094**	4.13 (2.07)	4.16 (2.29)	0.011	**3.13 (1.48)**	**3.18 (1.61)**	**0.034**	3.10 (1.46)	3.12 (1.53)	0.007
SAPII	48.91 (15.43)	49.68 (15.97)	0.049	48.84 (15.69)	49.93 (16.17)	0.068	**37.55 (12.56)**	**38.95 (13.06)**	**0.11**	37.88 (13.30)	37.88 (12.51)	< 0.001
Charlson comorbidity index	**6.72 (3.06)**	**5.73 (2.93)**	**0.331**	6.52 (3.07)	6.54 (2.92)	0.005	**6.25 (3.10)**	**5.62 (3.02)**	**0.205**	5.80 (3.15)	5.80 (3.03)	0.001
Heart rate	**89.45 (17.43)**	**92.42 (17.61)**	**0.17**	89.98 (17.40)	89.53 (17.28)	0.026	**87.67 (16.65)**	**86.43 (16.18)**	**0.076**	86.41 (16.46)	86.75 (15.78)	0.021
Temperature (℃)	36.84 (0.73)	36.85 (0.88)	0.01	36.86 (0.76)	36.88 (0.77)	0.023	**36.92 (0.50)**	**36.99 (0.61)**	**0.127**	36.99 (0.54)	37.01 (0.56)	0.021
MAP	**72.41 (8.23)**	**74.97 (8.71)**	**0.303**	72.87 (8.19)	72.67 (8.16)	0.026	**76.88 (11.16)**	**78.74 (10.35)**	**0.173**	77.99 (11.05)	78.08 (10.20)	0.008
WBC	14.81 (8.51)	15.23 (10.60)	0.043	14.91 (8.46)	14.52 (8.36)	0.047	**12.54 (10.40)**	**13.10 (8.56)**	**0.06**	12.52 (9.53)	12.78 (7.07)	0.031
Hemoglobin	**10.46 (2.13)**	**10.77 (2.10)**	**0.148**	10.57 (2.14)	10.59 (2.17)	0.013	**10.50 (2.10)**	**10.88 (2.03)**	**0.185**	10.76 (2.10)	10.80 (2.01)	0.021
Hematocrit	32.41 (6.36)	32.60 (6.33)	0.029	32.66 (6.38)	32.68 (6.65)	0.004	**32.07 (6.21)**	**32.76 (5.87)**	**0.116**	32.63 (6.13)	32.71 (5.86)	0.013
Platelet	190.44 (111.59)	189.30 (105.59)	0.01	192.79 (113.69)	187.79 (105.89)	0.046	**207.63 (117.95)**	**217.74 (113.55)**	**0.087**	213.40 (116.22)	216.83 (115.60)	0.03
Sodium	**138.62 (6.12)**	**138.19 (5.05)**	**0.076**	138.85 (6.05)	138.66 (5.59)	0.031	**138.20 (5.70)**	**138.53 (4.81)**	**0.062**	138.56 (5.48)	138.54 (4.91)	0.004
Potassium	4.39 (0.70)	4.37 (0.63)	0.04	4.36 (0.69)	4.40 (0.66)	0.064	**4.21 (0.64)**	**4.19 (0.57)**	**0.035**	4.19 (0.61)	4.18 (0.58)	0.011
Bicarbonate	**20.92 (4.78)**	**20.30 (4.42)**	**0.134**	20.89 (4.73)	20.60 (4.88)	0.06	**23.30 (4.80)**	**23.46 (4.42)**	**0.036**	23.55 (4.49)	23.60 (4.51)	0.01
Chloride	**103.20 (7.54)**	**104.72 (6.41)**	**0.218**	103.75 (7.40)	103.68 (6.96)	0.009	**103.22 (6.82)**	**104.50 (5.94)**	**0.2**	104.10 (6.60)	104.09 (6.02)	0.002
BUN	**36.94 (25.15)**	**31.10 (22.57)**	**0.244**	35.35 (23.88)	36.20 (26.71)	0.033	**31.50 (24.61)**	**27.16 (22.02)**	**0.186**	27.40 (20.50)	27.53 (21.98)	0.006
Creatine	**1.91 (1.60)**	**1.75 (1.43)**	**0.107**	1.80 (1.37)	1.88 (1.51)	0.058	**1.65 (1.71)**	**1.44 (1.40)**	**0.13**	1.43 (1.40)	1.44 (1.40)	0.009
Glucose	163.90 (79.44)	165.46 (69.44)	0.021	165.10 (81.18)	163.75 (70.01)	0.018	**143.22 (66.78)**	**146.34 (50.92)**	**0.053**	144.99 (75.73)	145.15 (49.96)	0.002
Anion gap	**17.40 (4.89)**	**16.81 (5.01)**	**0.119**	17.22 (4.78)	17.34 (4.92)	0.024	**15.14 (3.61)**	**14.41 (3.36)**	**0.209**	14.55 (3.32)	14.57 (3.35)	0.007
INR	**1.77 (1.01)**	**1.69 (0.90)**	**0.081**	1.73 (1.00)	1.74 (0.96)	0.012	**1.56 (0.90)**	**1.43 (0.73)**	**0.157**	1.46 (0.75)	1.46 (0.78)	0.006
PT	**19.24 (11.43)**	**18.30 (9.35)**	**0.09**	18.89 (11.40)	18.92 (10.21)	0.002	**16.88 (9.08)**	**15.60 (7.11)**	**0.157**	15.84 (7.37)	15.81 (7.52)	0.005
APTT	41.71 (20.36)	42.15 (19.80)	0.022	42.02 (20.43)	43.44 (21.24)	0.068	35.98 (16.19)	35.71 (16.59)	0.017	35.54 (15.70)	35.45 (15.48)	0.006
ICU LOS	**4.93 (4.28)**	**8.29 (8.28)**	**0.509**	**5.17 (4.48)**	**6.69 (6.90)**	**0.263**	**3.44 (3.12)**	**7.56 (7.92)**	**0.686**	**4.31 (3.94)**	**6.77 (7.23)**	**0.422**
Hospital LOS	**12.70 (13.74)**	**16.57 (17.22)**	**0.248**	**12.62 (13.79)**	**14.82 (21.96)**	**0.122**	**10.85 (11.07)**	**15.71 (15.35)**	**0.363**	**11.66 (11.04)**	**14.69 (13.39)**	**0.247**
	N (%)	N (%)	SMD	N (%)	N (%)	SMD	N (%)	N (%)	SMD	N (%)	N (%)	SMD
Female	**318 (45.8)**	**1,362 (40.2)**	**0.113**	276 (46.9)	266 (45.2)	0.034	**4,290 (46.6)**	**2,250 (42.4)**	**0.084**	1,498 (42.9)	1,524 (43.7)	0.015
Admission type (urgent/emergence)	565 (81.4)	2,844 (84.0)	0.07	486 (82.5)	494 (83.9)	0.036	**7,894 (85.7)**	**4,302 (81.1)**	**0.125**	2,890 (82.8)	2,878 (82.5)	0.009
Ethnicity (white)	428 (61.7)	2,118 (62.6)	0.019	355 (60.3)	358 (60.8)	0.01	6,218 (67.5)	3,520 (66.4)	0.025	2,289 (65.6)	2,290 (65.6)	0.001
First care unit												
MICU	**274 (39.5)**	**732 (21.6)**	**0.738**	221 (37.5)	172 (29.2)	0.024	**3,140 (34.1)**	**976 (18.4)**	**0.756**	1,145 (32.8)	698 (20.0)	0.016
SICU	**85 (12.2)**	**1,222 (36.1)**		78 (13.2)	196 (33.3)		**1,662 (18.1)**	**2,502 (47.2)**		726 (20.8)	1,651 (47.3)	
MICU/SICU	**194 (28.0)**	**442 (13.1)**		169 (28.7)	96 (16.3)		**2,961 (32.2)**	**764 (14.4)**		1,041 (29.8)	475 (13.6)	
Other	**141 (20.3)**	**988 (29.2)**		121 (20.5)	125 (21.2)		**1,443 (15.7)**	**1,061 (20.0)**		577 (16.5)	665 (19.1)	
CHF	**262 (37.8)**	**1,018 (30.1)**	**0.163**	207 (35.1)	223 (37.9)	0.056	**2,950 (32.0)**	**1,479 (27.9)**	**0.091**	974 (27.9)	995 (28.5)	0.013
Renal disease	**178 (25.6)**	**650 (19.2)**	**0.155**	140 (23.8)	147 (25.0)	0.028	**2,428 (26.4)**	**1,076 (20.3)**	**0.144**	742 (21.3)	738 (21.2)	0.003
COPD	182 (26.2)	832 (24.6)	0.038	157 (26.7)	167 (28.4)	0.038	2,618 (28.4)	1,452 (27.4)	0.024	962 (27.6)	964 (27.6)	0.001
Malignancy	**149 (21.5)**	**543 (16.0)**	**0.139**	123 (20.9)	124 (21.1)	0.004	**1,894 (20.6)**	**887 (16.7)**	**0.099**	625 (17.9)	640 (18.3)	0.011
Liver disease	189 (27.2)	850 (25.1)	0.048	159 (27.0)	171 (29.0)	0.045	**1,496 (16.3)**	**770 (14.5)**	**0.048**	558 (16.0)	516 (14.8)	0.033
CRRT	**31 (4.5)**	**591 (17.5)**	**0.425**	30 (5.1)	32 (5.4)	0.015	**44 (0.5)**	**306 (5.8)**	**0.308**	39 (1.1)	42 (1.2)	0.008
MV	**420 (60.5)**	**3,018 (89.2)**	**0.7**	412 (70.0)	422 (71.6)	0.037	**2,312 (25.1)**	**3,748 (70.7)**	**1.025**	2,042 (58.5)	2,006 (57.5)	0.021
Sedative medication	**442 (63.7)**	**3,182 (94.0)**	**0.8**	438 (74.4)	448 (76.1)	0.039	**3,023 (32.8)**	**4,155 (78.4)**	**1.03**	2,384 (68.3)	2,354 (67.5)	0.018
In-hospital mortality	230 (33.1)	1104 (32.6%)	0.011	199 (33.8)	207 (35.1)	0.032	**1100 (11.9)**	**867 (16.3)**	**0.127**	**401 (11.5)**	**518 (14.8)**	**0.108**

*AC* Arterial catheterization, *SMD* Standardized mean differences, *SOFA* Sequential Organ Failure Assessment, *SAPII* Simplified Acute Physiology Score II, *MAP* Mean arterial pressure, *WBC* White blood cell, *BUN* Blood urea nitrogen, *INR* International normalized ratio, *PT* Prothrombin time, *APTT* Active partial thromboplastin time, *MICU* Medical intensive care unit, *SICU* Surgery intensive care unit, *CHF* Congestive heart failure, *COPD* Chronic pulmonary diseases, *CRRT* Continuous renal replacement therapy, *MV* Mechanical ventilation, *LOS* Length of stay. Bolded values indicate statistical significance

Notably, there was no significant difference in hospital mortality between AC and non-AC group in patients with septic shock (32.6% vs 33.1%, *p* = 0.791). On the contrary, the in-hospital mortality of AC patients was significantly higher than that of non-AC patients in sepsis without shock group (16.3% vs 11.9%, *p* < 0.001).

### Propensity score matching

PSM yielded 589 pairs of patients who did not have an AC placement and patients who had an AC placement in septic shock group. Another propensity-matched sample consisted of 3,489 pairs of patients in sepsis without shock group. The SMDs before and after match were shown in Table 1 and Figure S2. After PSM, all covariates achieved balance (SMD < 0.1).

After PSM, there was no significant difference in hospital mortality between AC (35.1%) and non-AC groups (33.8%) in patient with septic shock (OR, 1.06; 95% CI, 0.84**–**1.35; *p* = 0.624) (Tables 1 and 2). However, the lengths of ICU stay (6.69 vs 5.17, *p* < 0.001) and hospital stay (14.82 vs 12.62, *p* = 0.040) of AC group were longer than those of non-AC group. For patients in sepsis without shock group, the in-hospital mortality of AC group (14.8%) was still higher than that of non-AC group (11.5%) even after well matching of baseline characteristics (OR, 1.34; 95% CI, 1.17**–**1.54; *p* < 0.001). Similarly, the lengths of ICU stay (6.77 vs 4.31, *p* < 0.001) and hospital stay (14.69 vs 11.66, *p* < 0.001) of AC group were also longer than those of non-AC group.

**Table 2:** ORs for In-hospital Mortality Associated with AC Placement for Sepsis with or without Shock Patients

Method	All patients with AC placement				Patients with AC placement within 24 h			
	Septic shock		Sepsis without shock		Septic shock		Sepsis without shock	
	OR (95% CI)	*P* Value	OR (95% CI)	*P* Value	OR (95% CI)	*P* Value	OR (95% CI)	*P* Value
Propensity score matching	1.06 (0.84—1.35)	0.624	1.34 (1.17—1.54)	< 0.001	0.99 (0.78 – 1.27)	0.950	1.08 (0.93 – 1.27)	0.306
Propensity score IPTW	1.05 (0.86 -1.28)	0.654	1.33 (1.18—1.50)	< 0.001	0.97 (0.79 – 1.20)	0.805	1.15 (1.00 – 1.31)	0.044
Multivariable logistic regression	0.99 (0.78—1.26)	0.960	1.37 (1.20—1.57)	< 0.001	0.93 (0.74 – 1.16)	0.518	1.14 (0.99 – 1.31)	0.061

Multivariable logistic regression was adjusted for age, gender, weight, admission type, ethnicity, first care unit, SOFA score, SAPII, Charlson Comorbidity Index, CHF, renal disease, COPD, malignancy, liver disease, heart rate, temperature, MAP, WBC count, hemoglobin, hematocrit, platelet, sodium, potassium, bicarbonate, chloride, BUN, creatine, glucose, anion gap, INR, PT, APTT, receipt of CRRT, MV, and sedative medication. IPTW, inverse probability of treatment weighting. *P* < 0.0013 were considered statistically significant

Subsequently, we repeated the above analysis in patients with AC placement within 24 h after ICU admission. 7248 patients were placed within 24 h after ICU admission, including 4240 patients without septic shock and 3008 patients with septic shock. As Table 2 showed, in patients with septic shock, we can draw the same conclusion that AC was not associated with improvements in hospital mortality. However, in hemodynamically stable sepsis patients, AC showed a tendency to be harmful while there were not statically significant.

### Sensitivity analyses

We performed additional modeling analyses using logistic regression and propensity score IPTW model, yielding similar results: there was no association between AC placement and in-hospital mortality in patients with septic shock, while AC placement was associated with increased in-hospital mortality in sepsis without shock group (Table 2).

**Fig. 2:**
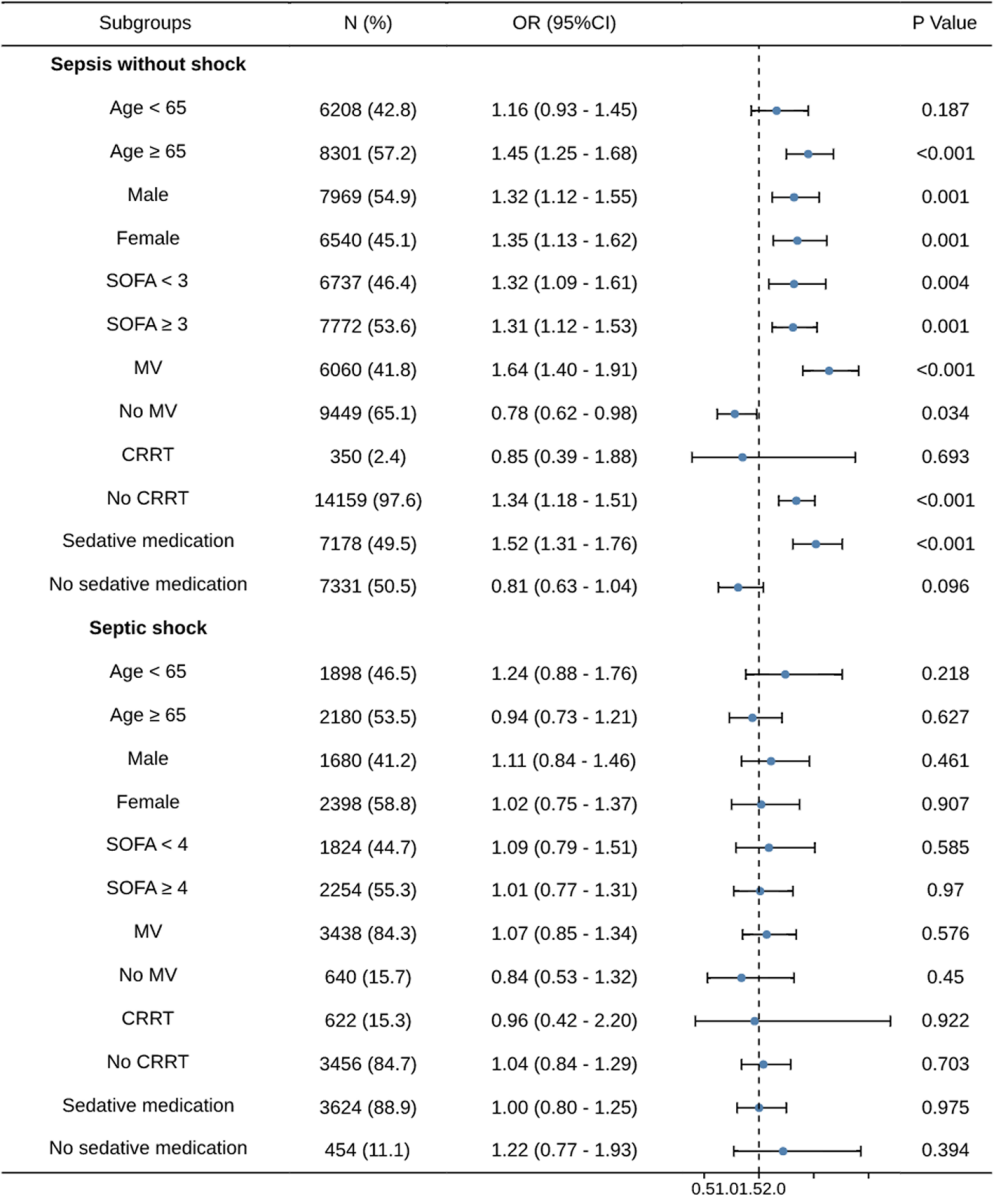
Odds Ratios (95% CIs) for In-hospital Mortality Associated with AC Placement in Subgroups. All odds ratios were derived from IPTW. MV, mechanical ventilation, CRRT, continuous renal replacement therapy

The impact of AC placement on subgroups classified according to age, gender, median of SOFA score, use of MV, CRRT, and sedative medication was shown in Table S1 and Fig. 2 showed the results of IPTW in the form of forest plot. For patients with septic shock, the results of all subgroups reported no association between AC placement and in-hospital mortality.

However, for the sepsis without shock group, not all subgroups yielded consistent results. AC placement was associated with higher in-hospital mortality in patients aged ≥ 65, using MV and sedative medication, and patients without CRRT. In addition, for gender subgroups or patients with SOFA score < 3, the results of propensity score IPTW model showed higher mortality in AC group than non-AC group while the result of logistic regression and PSM was not statistically significant. Finally, for patients age < 65, SOFA < 3, using CRRT, and patients without MV or sedative medication, all results supported no correlation between AC placement and in-hospital mortality.

## Discussions

An intervention should be considered only when it will provide benefits. There have been several examples of interventions widely used in previous clinical practice, which were proven to be of no benefit, some were even harmful, such as intracranial pressure monitors for patients with severe traumatic brain injury [14] and low-dose dopamine in renal failure [15]. Another example is pulmonary arterial catheters, a physiologic monitoring device, which was proven to be non-beneficial for all subgroups of critically ill patients after 14 subsequent randomized clinical trials [16].

AC is now routinely used in ICU. It was believed to provide immediate and reproducible measurement of BP [3]. However, evidence showed that a hyper-resonant blood pressure trace, derived from AC, significantly overestimates true systolic blood pressure and underestimates the diastolic pressure [17]. From this perspective, AC is also prone to be inaccurate. Localized complications of AC include limb ischemia, temporary occlusion (19.7%), bleeding, hematoma (14.4%), pseudoaneurysm, and local infection [6]. They have an incidence close to 11 per 1000 arterial catheter days. Besides, AC is a major cause of bloodstream infection, whose incidence is 1.7 per 1000 (95% CI: 1.2 to 2.3), 2.5-fold higher than peripheral intravenous catheters [18].

In this propensity-matched cohort analysis, we reported no association between AC use and in-hospital mortality in patients with septic shock. Nevertheless, in sepsis without shock group, the AC use may be associated with higher in-hospital mortality.

There was no other study that reported beneficial outcomes associated with AC. Similarly, one observational study performed on the Project IMPACT database showed that ACs were not associated with improvements in hospital mortality in critically ill patients [19]. In the cohort of patients receiving vasopressors, the odds of death were increased in patients with AC use (OR, 1.08; 95% CI, 1.02**–**1.14; *P* = 0.008). Another large cohort focusing on ventilated patients without vasopressor support, demonstrated no difference in day 28 mortality between patients with and without AC after PSM [20].

In this study, for the primary cohort (Table 2) and all of the secondary cohorts (Fig. 2, Table S1) in septic shock group, we found no association between AC placement and outcomes. One potential interpretation is that the AC use does not bring net mortality benefit to septic shock patients. The blood gas testing and hemodynamic monitoring obtained from IAC don’t translate into effects on mortality; or the risks it brings offset its benefits. Alternatively, the results of this analysis may be attributed to unmeasured confounding, despite attempts to adjust for confounders by using multiple logistic regression and PSM. Patients receiving ACs are potentially in more severe condition, which may not be able to be distinguished by current indicators and severity scores. The use of ACs ameliorates and covers this imbalance, thus no mortality effect is detected.

As for sepsis without shock group, both the primary cohort (Table 2) and 4 of 10 secondary cohorts (Fig. 2) demonstrated that AC was associated with increased risk of mortality. Hemodynamically stable patients are less likely to benefit from AC, while the use of AC will increase the risk of complications, which might lead to higher mortality. In addition, AC placement seemed to be harmful in hemodynamically stable sepsis patients with AC placement with 24 h after ICU admission while some results showed no statistical difference. On the one hand, it may be that the AC placement does not have a significant impact on in-hospital mortality, and the harmful result in the primary cohort may come from the bias caused by the measure time of patient characteristics. On the other hand, it may be caused by the small number of patients. Overall, AC placement did not show beneficial effect on hemodynamically stable sepsis patients.

There are several limitations in the present study that should be considered. First, as mentioned above, residual confounding can never be eliminated in retrospective studies, although we attempted to account for this through replicating results across multiple analyses for the primary cohort and multiple secondary cohorts. Second, this is a non-random, single-center study. Our conclusion may not be applicable to other institutions, and causality between AC and mortality cannot be established from this study. It raises the need for replication in randomized controlled trials to evaluate this topic. Third, potential adverse events associated with AC are not available in MIMIC database. It cannot be investigated thoroughly whether the AC placement increases the risk of catheter-associated bloodstream infections or vascular complications in sepsis. Lastly, the baseline SOFA score is assumed to be zero, as we do not know if the patient has preexisting (acute or chronic) organ dysfunction before the onset of infection. Although this may lead to the inclusion of some unqualified cases, to our knowledge, this is a widely recognized method for identifying patients with sepsis in this database [21].

## Conclusion

Our results suggested that AC placement did not improve the survival of patients with septic shock; on the contrary, it may increase in-hospital mortality in hemodynamically stable septic patients. Besides, AC was associated with an increased ICU LOS and hospital LOS. These results highlight the need for randomized controlled trials to investigate the impact of AC use on patient outcomes.

## Supplementary Information

**Additional file 1: Figure S1.** Summary of Missing Data of Covariates.

**Additional file 2: Figure S2.** Standardized Mean Differences before and after Match.

**Additional file 3: Table S1.** Odds Ratios (95% CIs) for In-hospital Mortality Associated with AC Placement in Subgroups.

## Data Availability

The datasets used and analyzed during the current study are available on MIMIC website (https://physionet.org/content/mimiciv/1.0/). The code used in this article can be found in https://github.com/MIT-LCP/mimic-iv/tree/master/concepts.
