# Supplementary figures and images for: Arterial catheterization and in-hospital mortality in sepsis: a propensity score-matched study
Source: BMC Anesthesiol. 2022 Jun 9;22:178. doi: 10.1186/s12871-022-01722-5 (PMC9178844; doi:10.1186/s12871-022-01722-5)

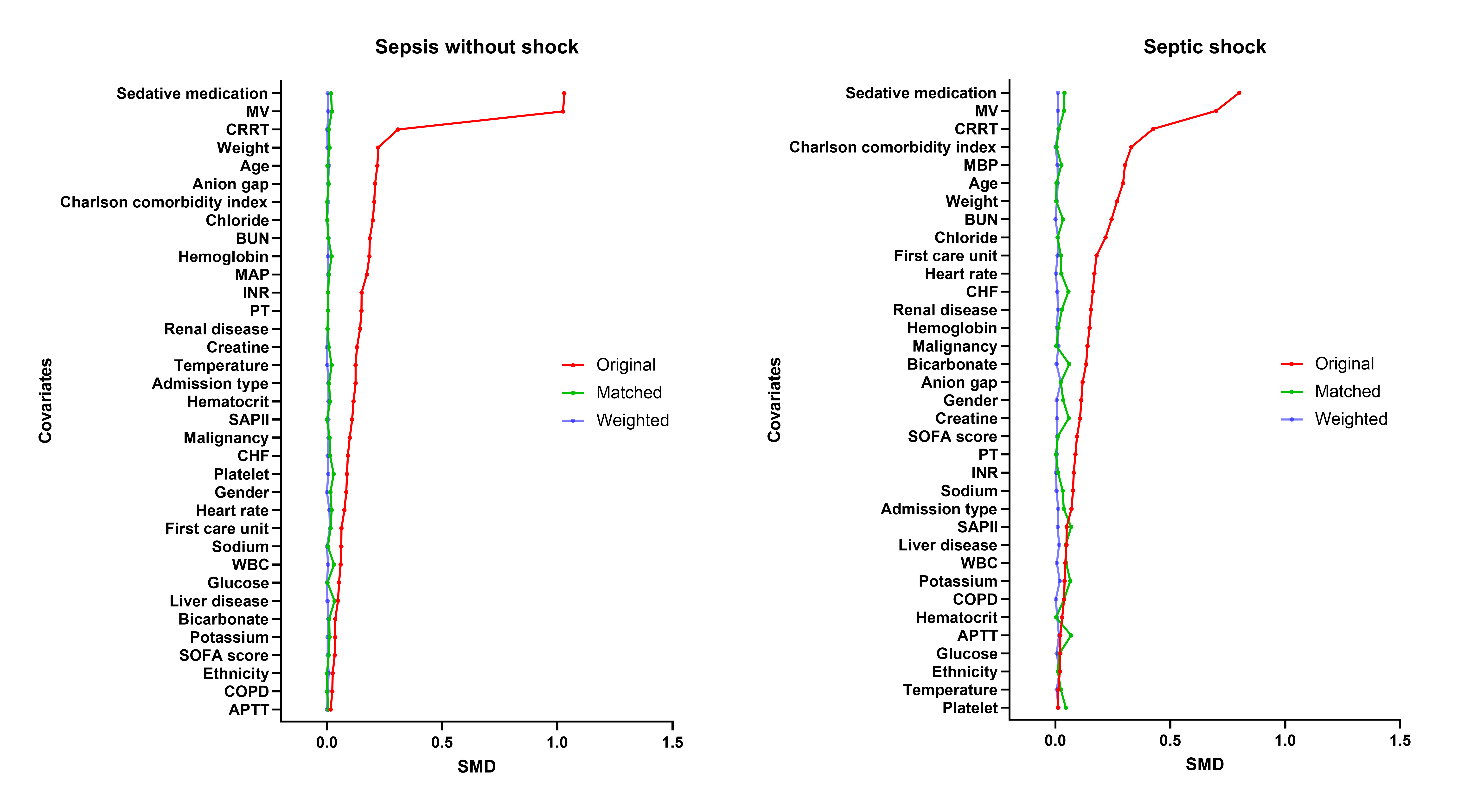

Supplement: Supplementary file 2 — Additional file 2: Figure S2. Standardized Mean Differences before and after Match. [file 12871_2022_1722_MOESM2_ESM.tif]
