# Supplementary material for: Arterial catheterization and in-hospital mortality in sepsis: a propensity score-matched study
Source: BMC Anesthesiol. 2022 Jun 9;22:178. doi: 10.1186/s12871-022-01722-5 (PMC9178844; doi:10.1186/s12871-022-01722-5)
Supplement: Supplementary file 3 — Additional file 3: Table S1. Odds Ratios (95% CIs) for In-hospital Mortality Associated with AC Placement in Subgroups. [file 12871_2022_1722_MOESM3_ESM.docx]

**Table S1.** Odds Ratios (95% CIs) for In-hospital Mortality Associated with AC Placement in Subgroups.

| Subgroups | Matched | | Weighted | | Multivariable Logistic Regression | |
| --- | --- | --- | --- | --- | --- | --- |
|  | OR (95% CI) | P Value | OR (95% CI) | P Value | OR (95% CI) | P Value |
| Septic shock |  | |  | |  |  |
| Age < 65 | 1.10 (0.72**–**1.67) | 0.669 | 1.24 (0.88**–**1.76) | 0.218 | 1.23(0.83-1.82) | 0.295 |
| Age ≥ 65 | 0.94 (0.70**–**1.27) | 0.705 | 0.94 (0.73**–**1.21) | 0.627 | 0.97(0.73-1.28) | 0.809 |
| Male | 1.07 (0.77**–**1.50) | 0.673 | 1.11 (0.84**–**1.46) | 0.461 | 1.07(0.79-1.45) | 0.677 |
| Female | 0.88 (0.62**–**1.25) | 0.47 | 1.02 (0.75**–**1.37) | 0.907 | 0.98(0.71-1.37) | 0.926 |
| SOFA < 4 | 0.95 (0.65**–**1.38) | 0.771 | 1.09 (0.79**–**1.51) | 0.585 | 1.07(0.76-1.52) | 0.688 |
| SOFA ≥ 4 | 0.97 (0.71**–**1.33) | 0.873 | 1.01 (0.77**–**1.31) | 0.97 | 1.03(0.77-1.39) | 0.829 |
| No MV | 0.77 (0.46**–**1.27) | 0.305 | 0.84 (0.53**–**1.32) | 0.45 | 0.81(0.47-1.40) | 0.445 |
| MV | 1.02 (0.77**–**1.35) | 0.886 | 1.07 (0.85**–**1.34) | 0.576 | 1.08(0.84-1.39) | 0.547 |
| No CRRT | 1.00 (0.78**–**1.29) | 1 | 1.04 (0.84**–**1.29) | 0.703 | 1.01(0.80-1.28) | 0.940 |
| CRRT | 0.86 (0.29**–**2.54) | 0.783 | 0.96 (0.42**–**2.20) | 0.922 | 0.98(0.41-2.33) | 0.954 |
| No Sedative medication | 1.20 (0.71**–**2.05) | 0.498 | 1.22 (0.77**–**1.93) | 0.394 | 1.48(0.85-2.58) | 0.165 |
| Sedative medication | 1.02 (0.77**–**1.34) | 0.889 | 1.00 (0.80**–**1.25) | 0.975 | 0.97(0.75-1.24) | 0.787 |
| Sepsis without shock |  |  |  |  |  |  |
| Age < 65 | 1.11 (0.87**–**1.43) | 0.404 | 1.16 (0.93**–**1.45) | 0.187 | 1.20(0.95-1.51) | 0.133 |
| Age ≥ 65 | **1.41 (1.19–1.67)** | **<0.001** | **1.45 (1.25–1.68)** | **<0.001** | **1.36(1.17-1.58)** | **<0.001** |
| Male | 1.30 (1.07**–**1.57) | 0.008 | **1.32 (1.12–1.55)** | **0.001** | 1.28(1.08-1.51) | 0.005 |
| Female | 1.33 (1.08**–**1.63) | 0.007 | **1.35 (1.13–1.62)** | **0.001** | 1.33(1.10-1.61) | 0.003 |
| SOFA < 3 | 1.22 (0.98**–**1.52) | 0.081 | 1.32 (1.09**–**1.61) | 0.004 | 1.31(1.07-1.60) | 0.008 |
| SOFA ≥ 3 | 1.29 (1.08**–**1.54) | 0.005 | **1.31 (1.12–1.53)** | **0.001** | 1.27(1.08-1.49) | 0.004 |
| No MV | 0.76 (0.57**–**1.00) | 0.054 | 0.78 (0.62**–**0.98) | 0.034 | 0.73(0.57-0.93) | 0.010 |
| MV | **1.59 (1.34–1.88)** | **<0.001** | **1.64 (1.40–1.91)** | **<0.001** | **1.79(1.52-2.10)** | **<0.001** |
| No CRRT | **1.31 (1.14–1.51)** | **<0.001** | **1.34 (1.18–1.51)** | **<0.001** | **1.32(1.16-1.50)** | **<0.001** |
| CRRT | 1.00 (0.39**–**2.59) | 1 | 0.85 (0.39**–**1.88) | 0.693 | 0.96(0.38-2.42) | 0.927 |
| No Sedative medication | 0.83 (0.61**–**1.13) | 0.24 | 0.81 (0.63**–**1.04) | 0.096 | 0.75 (0.58**–**0.98) | 0.035 |
| Sedative medication | **1.45 (1.23–1.70)** | **<0.001** | **1.52 (1.31–1.76)** | **<0.001** | **1.64 (1.40–1.91)** | **<0.001** |

Multivariable logistic regression was adjusted for age, gender, weight, admission type, ethnicity, first care unit, SOFA score, SAPII, Charlson Comorbidity Index, CHF, renal disease, COPD, malignancy, liver disease, heart rate, temperature, MAP, WBC count, hemoglobin, hematocrit, platelet, sodium, potassium, bicarbonate, chloride, BUN, creatine, glucose, anion gap, INR, PT, APTT, receipt of CRRT, MV, and sedative medication. SOFA score subgroups were divided according to the median value of each group. P < 0.0013 were considered statistically significant.
